# ATR-101, a selective ACAT1 inhibitor, decreases ACTH-stimulated cortisol concentrations in dogs with naturally occurring Cushing’s syndrome

**DOI:** 10.1186/s12902-018-0251-5

**Published:** 2018-05-02

**Authors:** Daniel K. Langlois, Michele C. Fritz, William D. Schall, N. Bari Olivier, Rebecca C. Smedley, Paul G. Pearson, Marc B. Bailie, Stephen W. Hunt

**Affiliations:** 1 Department of Small Animal Clinical Sciences, College of Veterinary Medicine, Michigan State University, East Lansing, MI 48824 USA; 2 Veterinary Diagnostic Laboratory, College of Veterinary Medicine, Michigan State University, East Lansing, MI 48824 USA; 3 Pearson Pharma Partners, Inc., Los Angeles, California 91362 USA; 4 Integrated Non-Clinical Development Solutions, Inc., Ann Arbor, MI 48103 USA; 5 Millendo Therapeutics, Inc., Ann Arbor, MI 48104 USA; 6 Present address: College of Human Medicine, Michigan State University, East Lansing, MI 48824 USA

**Keywords:** Hyperadrenocorticism, Adrenocortical carcinoma, Canine models of adrenal disease

## Abstract

**Background:**

Cushing’s syndrome in humans shares many similarities with its counterpart in dogs in terms of etiology (pituitary versus adrenal causes), clinical signs, and pathophysiologic sequelae. In both species, treatment of pituitary- and adrenal-dependent disease is met with limitations. ATR-101, a selective inhibitor of ACAT1 (acyl coenzyme A:cholesterol acyltransferase 1), is a novel small molecule therapeutic currently in clinical development for the treatment of adrenocortical carcinoma, congenital adrenal hyperplasia, and Cushing’s syndrome in humans. Previous studies in healthy dogs have shown that ATR-101 treatment led to rapid, dose-dependent decreases in adrenocorticotropic hormone (ACTH) stimulated cortisol levels. The purpose of this clinical study was to investigate the effects of ATR-101 in dogs with Cushing’s syndrome.

**Methods:**

ATR-101 pharmacokinetics and activity were assessed in 10 dogs with naturally-occurring Cushing’s syndrome, including 7 dogs with pituitary-dependent disease and 3 dogs with adrenal-dependent disease. ATR-101 was administered at 3 mg/kg PO once daily for one week, followed by 30 mg/kg PO once daily for one (*n* = 4) or three (*n* = 6) weeks. Clinical, biochemical, adrenal hormonal, and pharmacokinetic data were obtained weekly for study duration.

**Results:**

ATR-101 exposure increased with increasing dose. ACTH-stimulated cortisol concentrations, the primary endpoint for the study, were significantly decreased with responders (9 of 10 dogs) experiencing a mean ± standard deviation reduction in cortisol levels of 50 ± 17% at study completion. Decreases in pre-ACTH-stimulated cortisol concentrations were observed in some dogs although overall changes in pre-ACTH cortisol concentrations were not significant. The compound was well-tolerated and no serious drug-related adverse effects were reported.

**Conclusions:**

This study highlights the potential utility of naturally occurring canine Cushing’s syndrome as a model for human disease and provides proof of concept for ATR-101 as a novel agent for the treatment of endocrine disorders like Cushing’s syndrome in humans.

## Background

Hyperadrenocorticism, commonly known as Cushing’s syndrome (CS), is an endocrine disorder with many similarities between dogs and humans [1]. In both species, most cases of naturally-occurring disease result from adrenocorticotropic hormone (ACTH) secreting pituitary adenomas, while remaining cases often result from cortisol-secreting adrenal tumors [2, 3]. Regardless of etiology, clinical signs are usually a result of increased circulating cortisol concentrations. Dermatologic manifestations are frequent and can include thin skin, easy bruising, hyperpigmentation, and recurrent infections [4]. The catabolic effects of cortisol often lead to muscle wasting and central obesity resulting in a classic “Cushingoid” appearance. Serious consequences such as hypertension, dyslipidemias, and thromboembolism are reported in both species [2, 5].

In addition to pathophysiologic parallels, similar treatment options exist for both species [6–8]. However, the actual treatment pursued for pituitary-dependent disease often differs as hypophysectomy and irradiation, both common therapies for humans with pituitary lesions, are infrequently performed in dogs in part due to expense and limited availability. Furthermore, depending on definition used, procedural success of dogs undergoing hypophysectomy for CS could be as low as 60% when considering post-operative mortality, treatment failures, and disease relapse [7, 9]. Direct comparative studies have not been performed, but median survival times do not appear to be substantially different in dogs undergoing hypophysectomy compared to dogs receiving medical therapy alone [9–11]. As such, most dogs with Cushing’s disease are treated medically with agents that target the adrenal gland. Common therapies include trilostane, which inhibits enzymatic conversion of steroids by 3-beta-hydroxysteroid dehydrogenase, or mitotane, an adrenal cytotoxic agent [8, 12]. In veterinary medicine, the complicated dosing and side effects of mitotane have led to declining use, with a corresponding increase in trilostane treatment [13]. However, variable pharmacologic activity of trilostane has led to differing dosing recommendations with some dogs requiring up to three times daily dosing, and a small number of dogs having minimal response [14, 15]. Monitoring remains frequent, and fatal adrenal gland necrosis has been reported occasionally [15]. In humans, relapse of hypercortisolemia occurs in 15–66% of patients undergoing hypophysectomy, and persistent hypercortisolemia occurs in 14–72% of patients undergoing radiation therapy [2, 16]. Consequently, medical therapy is necessary for a subset of human patients; however, current options have limitations ranging from poor drug tolerability to variable efficacy [16].

Treatment has remained similar in dogs and humans with adrenal-dependent disease. Although adrenalectomy is curative for benign disease, management of malignant adrenocortical carcinoma (ACC) has remained challenging [17, 18]. Median survival times in dogs with detectable metastases are approximately 2–4 months despite medical or surgical intervention [18, 19]. In humans, surgical resection is palliative as the disease is often advanced at the time of diagnosis. Chemotherapeutics evaluated to date have been largely ineffective at reducing tumor burden, and controlling hypercortisolemia remains difficult [17, 20]. No drug is FDA approved for treatment of canine ACC, and the only FDA approved drug for treatment of human ACC is mitotane. However, stable disease or partial remissions only occur in approximately 30% of human patients with malignant disease, and survival times are poor [21]. Furthermore, mitotane toxicities are common and negatively affect quality of life in both humans and dogs [17, 20, 22]. Multi-agent protocols also have been used to treat human ACC; however, the median progression free survival of 5 months is discouraging [23].

Given these limitations, there is a critical need for new, targeted therapies for CS and ACC. ATR-101 (also known as PD132301–2) is a small molecule therapeutic in human clinical development for the treatment of ACC, congenital adrenal hyperplasia, and Cushing’s syndrome (CS). ATR-101 is a potent (IC_50_ = 0.009 μM) and selective inhibitor of acyl coenzyme A:cholesterol acyltransferase isoform 1 (ACAT1). ACAT1 catalyzes cholesterol ester formation from cholesterol and long-chain fatty acyl-CoA [24], and in the adrenal cortex, is particularly important in creating a reservoir of substrate for steroid biosynthesis. Inhibition of this enzyme by ATR-101 at low doses results in decreases in circulating cortisol levels and other adrenal steroids in a time- and dose-dependent manner due to the lack of esterified cholesterol reservoirs. At high doses, ATR-101 leads to accumulation of free cholesterol in adrenocortical cells, ultimately leading to cellular stress and apoptosis [25–27]. As such, ATR-101 therapy could be of benefit for multiple diseases associated with adrenal steroid dysregulation [26, 27]. Recent in vivo pharmacologic studies in healthy dogs [25, 27] have provided support for development of ATR-101 in ACC and endocrine diseases such as CS and congenital adrenal hyperplasia given that ATR-101 preferentially distributes to the adrenal glands and selectively inhibits adrenal ACAT1 activity. The frequently encountered gastrointestinal and neurologic side effects of mitotane have not been observed in initial studies of ATR-101 administration to healthy dogs [27]. A recent study has suggested that ACAT1 may also be a target of mitotane [28], but other, yet to be elucidated targets, may account for some of the effects of mitotane, although this has not been thoroughly investigated [29].

Given the similarities in dogs and humans with naturally occurring CS, the dog offers a unique opportunity to perform proof of concept clinical studies for a human therapeutic. In this study, the pharmacokinetics of ATR-101 were determined in dogs with naturally occurring CS. Furthermore, we investigated the biochemical and adrenal hormonal effects of ATR-101 administration over a 2–4 week time period. Adrenal gland histology was evaluated in dogs with adrenal-dependent disease. The results reported herein further support the ongoing development of ATR-101 as a novel agent for treatment of endocrine disorders associated with adrenal steroid dysregulation.

## Methods

### Compound

ATR-101 [N-[2,6-bis(1-methylethyl)phenyl]-N^′^-[[1-[4-(dimethylamino)phenyl]cyclopentyl]methyl]urea, hydrochloride salt (PD132301–2)] was synthesized by PharmAgra Labs, Inc. (Brevard, NC) using a modification of previously described methods [30]. The ATR-101 (Lot 313PAL23) used in these experiments had a purity of 95.7%. ATR-101 was compounded into gelatin capsules using microcrystalline cellulose as an excipient to meet target doses (3 mg/kg and 30 mg/kg) based on individual subject weights.

### Sample size calculation

A study of a continuous response variable (post-ACTH-stimulated cortisol concentrations following initial treatment) from matched pairs of study subjects was planned using previous data from dogs treated for CS at the Michigan State University Veterinary Medical Center. In order to detect a 35% decrease in post-ACTH-stimulated cortisol concentrations, 10 subjects would be needed to be able to reject the null hypothesis that this response difference is zero with probability (power) 0.8. The Type I error probability associated with this test of this null hypothesis is 0.05.

### Animal study

This study was a prospectively designed proof-of-concept study to evaluate the activity of ATR-101 in dogs with CS. Dogs with both pituitary and adrenal-dependent CS were included in the study given the clinical, hormonal, and biochemical similarities [3] coupled with current veterinary practice standards in which drugs that target the adrenal glands are used for medical management of both forms of disease [12, 19]. Considering the mechanistic features of ATR-101 [27], treatment would be expected to be of benefit for naturally occurring CS independent of etiology. Client-owned dogs with clinical signs, biochemical evidence, or a recent diagnosis of CS were recruited from the local veterinary community. All dogs underwent initial evaluation at the Michigan State University Veterinary Medical Center to include a complete patient history, thorough physical examination, complete blood count, serum biochemical profile, and urinalysis. Dogs with concurrent disease that could account for clinical signs or dogs that received any medications within the preceding 3 months that could alter endogenous cortisol production were excluded from participation. If suspicion for CS still existed following initial evaluation, an ACTH stimulation test or a low-dose dexamethasone suppression test (LDDST) were performed for confirmation [20, 31]. Dogs with confirmed CS underwent complete abdominal ultrasound evaluation and measurement of endogenous ACTH concentrations to determine if disease etiology was pituitary or adrenal in origin [20]. Dogs were classified as having pituitary-dependent disease if normal to increased endogenous ACTH concentrations (> 9 pmol/L; reference interval, 6.7–25.0 pmol/L) were detected coupled with ultrasound observation of approximately symmetric, normal to enlarged adrenal glands. Dogs were classified as having adrenal-dependent disease if decreased endogenous ACTH concentrations (< 4 pmol/L) were detected coupled with ultrasound observation of one irregularly enlarged adrenal gland and an atrophied contralateral adrenal gland. All dogs underwent ACTH stimulation testing within one week of study commencement to serve as baseline.

All dogs enrolled in the trial received ATR-101 at an initial once daily dose of 3 mg/kg PO for one week, followed immediately by a once daily dose of 30 mg/kg PO for either one week (*n* = 4) or 3 weeks (*n* = 6). The doses selected were based on previous pharmacologic and toxicologic investigations in healthy dogs [27]. The initial 4 dogs enrolled in the study received the 30 mg/kg dose for 1 week to ensure that drug was well-tolerated. Following an interim analysis in which adverse effects were not identified, the protocol was modified to extend ATR-101 treatment to assess if any additional biochemical, adrenal hormonal, or clinical effects would be observed with a longer duration of treatment. In this out-patient study, owners were instructed to administer ATR-101 capsules in the morning, preferably on an empty stomach, or with a small amount of food, if necessary for compliance. Capsules were administered by hospital technicians on study evaluation days. Dog owners recorded medication administration and any observed clinical changes or adverse effects on a standardized medication log. Weekly physical examinations and laboratory evaluations were performed in all dogs until study completion. Blood samples for laboratory evaluations were collected via peripheral venipuncture. Following study completion, owners of dogs with pituitary-dependent disease were instructed to allow a 4 week washout period before initiating standard medical therapies for CS such as trilostane. Dogs with adrenal-dependent disease underwent surgical adrenalectomy one day following study completion. All dogs remained housed with owners for the study duration.

### Measurement of ATR-101 concentrations

ATR-101 concentrations were measured in serum samples collected immediately before (time 0) and 1, 2, 4 and 8 h after ATR-101 administration on days 1 (*n* = 4), 7 (*n* = 10), 14 (*n* = 10), and 28 (*n* = 5). Concentrations of ATR-101 in serum were determined by a liquid chromatography coupled with tandem mass spectrometry (LC/MS-MS) method which was developed and validated by AIT Bioscience (Indianapolis, IN) for the quantitation of ATR-101 in K_2_EDTA dog plasma and has been described elsewhere [27]. This method was developed to cover the range of 1.00–1000 ng/mL of ATR-101, using ^13^C_4_-ATR-101 as the internal standard. Samples were analyzed on a Waters Acquity UPLC™ liquid chromatograph interfaced with a Thermo Scientific TSQ Vantage triple quadrupole mass spectrometer with electrospray ionization in the positive ion mode. ATR-101 was detected by selected reaction monitoring of the m/z 422 → 202 transition and its internal standard was detected by selected reaction monitoring of the m/z 426 → 206 transition. Raw data from the mass spectrometer was acquired and processed in Thermo Scientific Watson Laboratory Information Management System (LIMS). Peak area ratios from the calibration standard responses were regressed using a (1/concentration^2^) linear fit for ATR-101.

### Pharmacokinetic analysis

Serum ATR-101 concentrations were analyzed by noncompartmental analysis (NCA) with Phoenix™ WinNonlin^®^ Version 6.3, using an extravascular administration model. Nominal doses and sampling times were used. Prior to T_max_, values that were below the lower limit of quantitation (BLQ) were set to 0; other BLQ concentrations were excluded from the analysis. The area under the curve from time zero to the last measurable concentration (AUC_0-t_) was calculated using the linear up/log down method. Log/linear regression through the last three or more time points (excluding T_max_) was used to estimate the elimination constant (λ_z_). The apparent terminal phase half-life (T_1/2_) and the AUC from time zero to infinity (AUC_0-∞_) were calculated using the following equations: T_1/2_ = ln (2)/λ_z_, *and* AUC_0 − ∞_ = AUC_0 − *t*_ + C_t, pred_/*λ*_*z*_, where C_t,pred_ is the last predicted concentration based on the exponential decline (e.g., e^-λz·t^). If any of the following were observed, the terminal phase-dependent parameters (e.g., λ_z_, T_1/2_, AUC_0-∞_, CL/F, V_z_/F) following single dose administration were not reported: λ_z_ indicated a positive slope (λ_z_ > 0); T_max_ was one of the last three time points with measurable concentrations; or the linear regression coefficient or the goodness of fit (R^2^) was less than 0.80. This also applied to λ_z_, T_1/2_, AUC_0-∞_, and V_z_/F at steady state. Mean concentration graphs were prepared with Prism™ Version 7.0 (GraphPad Software, Inc., La Jolla, CA). If less than 50% of the animals had reportable concentrations (e.g., 24 h post dose), the means were not plotted.

### Measurement of routine hematologic and serum biochemical parameters

Baseline and weekly assessment of hematologic and biochemical parameters occurred for the study duration in all dogs. Blood samples obtained via peripheral venipuncture were divided equally into a K_2_EDTA plasma and a serum collection tube. Plasma and serum (following clot formation) were harvested from the collection tubes immediately following centrifugation at 1200 X *g* for 10 min at 4 °C. Routine whole blood hematologic and serum biochemical analyses were then performed using an Advia 120 Hematology System (Siemens Healthcare, Deerfield, IL) and an Olympus AU640^e^, (Olympus America Inc., Center Valley, PA), respectively. Weekly determinations of urine specific gravity were performed on voided urine samples using a standard veterinary refractometer (Reichert TS meter, 10,406, Cambridge Instruments Inc., Buffalo, NY).

### Adrenal function testing

All dogs underwent baseline and weekly ACTH stimulation testing. This test consists of plasma sampling for cortisol and aldosterone concentrations immediately before and one hour post-administration of 5 μg/kg synthetic ACTH (Cortrosyn, Amphastar Pharmaceuticals Inc., Rancho Cucamonga, CA) intravenously. Although less sensitive than the LDDST for diagnosing CS, this dynamic adrenal function assessment offers the greatest specificity of available diagnostic tests, it offsets the variability in single point measurements, and it is the standard method for monitoring the efficacy of medical therapy [20, 31, 32]. Testing was performed in the morning immediately following ATR-101 administration.

### Measurement of cortisol, aldosterone, and ACTH concentrations

Blood samples obtained via peripheral venipuncture were placed into K_2_EDTA collection tubes, and plasma was harvested immediately after centrifugation at 1200 X *g* for 10 min at 4 °C. Radioimmunoassay kits previously validated and utilized for clinical diagnostics and research at the Michigan State University Diagnostic Center for Population and Animal Health were used to measure plasma concentrations of cortisol (Coat-a-Count Cortisol, Siemens Medical Solutions Diagnostics, Los Angeles, CA) [33], aldosterone (Coat-a-Count Aldosterone, Siemens Medical Solutions Diagnostics, Los Angeles, CA) [34], and ACTH (ACTH Immunoradiometric Assay, Scantibodies Laboratory, Inc., Santee, CA) [35].

### Adrenal gland histology and immunohistochemistry

Following adrenalectomy, adrenal gland specimens were fixed in 10% buffered formalin for 24 h and then routinely trimmed, embedded, processed, and stained with hematoxylin and eosin for histologic evaluation. Previous established criteria were used to classify lesions as adenomas or carcinomas [36]. In addition, immunohistochemical (IHC) labeling for Melan-A and inhibin (Dako, Carpinteria, CA, USA) was performed on 5 μm serial sections of two of the three submitted adrenal gland specimens, and immunolabeling for caspase-3 (RDI/Fitzgerald, Acton, MA, USA), was performed on two of the three adrenal gland specimens [37, 38]. Immunolabeling for inhibin was performed on a Leica Bond-Max autostainer (Leica Microsystems, Buffalo Grove, IL, USA). Antigen retrieval was performed using ER1 epitope for 20 min in citric buffer solution on-line retrieval. Sections were incubated with a mouse monoclonal primary antibody (#M3609, DAKO, Carpinteria, CA, USA) against inhibin at a dilution of 1:100. A streptavidin-biotin labeling system (polymer: Refine Detection, Leica Microsystems, Buffalo Grove, IL, USA) was used for immunolabeling, and reactions were visualized with 3,3′-diaminobenzidine. Immunolabeling for Melan-A and caspase-3 was performed on an Ultra autostainer (Ventana Medical Systems, Tucson, AZ, USA). Antigen retrieval for Melan-A was performed using PT Link, citric buffer solution for 60 min, DAKO, Carpinteria, CA, USA). Antigen retrieval for caspase-3 was performed using CC1 Standard high pH for 20 min (on-line retrieval) epitope retrieval. Sections of adrenal gland were incubated for 30 min with 1 of the following 2 primary antibodies: mouse monoclonal anti-Melan-A (A103 clone Dako M7196, Carpinteria, CA, USA) at a dilution of 1:20 or rabbit polyclonal anti-caspase-3 (# 20RCR013, RDI/Fitzgerald, Acton, MA, USA) at a dilution of 1:1000. All slides were counterstained with hematoxylin. Sections of normal canine adrenal gland were used as positive controls for inhibin and Melan-A; sections of canine lymph node were used as positive controls for caspase-3.

### Statistical analysis

Data were reported as means ± standard deviations (SD) given normal distribution as assessed by Kolmogorov-Smirnov testing and boxplot analysis. For analysis of pharmacokinetic parameters, C_max_, AUC, half-life, oral clearance (CL/F), and volume of distribution (Vz/F) at different dosages were compared using a two-tailed Student’s *t-*test. The effect of treatment over time on cortisol and aldosterone concentrations and other clinically pertinent laboratory data were evaluated using a repeated measures analysis of variance. When a significant effect for time was detected, a Dunnett’s post hoc test was performed to compare individual treatment values to pre-treatment control. For the cortisol comparisons, the tests were conducted in a one-tailed fashion based on the prediction that cortisol values would decline. All other comparisons were made in two-tailed fashion. Differences were considered significant at *P* ≤ 0.05. Statistical analyses were performed using commercially available software (GraphPad Prism™ Version 7.0, Graphpad Software Inc., La Jolla, CA; and Statistica, Dell StatSoft Inc., Austin, TX).

## Results

### Animal demographics

Of 15 dogs screened for potential study participation, 10 dogs with naturally occurring CS, based on adrenal function testing, were enrolled in the clinical trial (Table 1). One dog (dog 9) was withdrawn at day 24 due to complications from an unrelated prostatic carcinoma; data through day 21 from this subject were still used for analysis. Dogs were classified as having pituitary- or adrenal-dependent disease on the basis of abdominal ultrasound examination and measurement of endogenous ACTH concentration [20]. Overall, 7 dogs were classified as having pituitary-dependent disease, while 3 were classified as adrenal-dependent. In 3 dogs (dogs 1, 8, 10), CS sub-type initially was unclear. The plasma endogenous ACTH concentrations of 5.1 pmol/L, 8.8 pmol/L, and 8.7 pmol/L for dogs 1, 8, and 10, respectively, were deemed equivocal as they were near the low-end of the reference interval (6.7–25.0 pmol/L). Results of abdominal ultrasound examination were definitive in all three cases (bilaterally symmetric, normal to mildly enlarged adrenal glands in 2 dogs with pituitary dependent disease; one enlarged, irregular adrenal gland with atrophy of the contralateral adrenal gland in 1 dog with adrenal dependent disease), and used for definitive classification. Results of measured endogenous ACTH concentrations and abdominal ultrasound examinations were in agreement for remaining dogs.

**Table 1:** Demographics of the 10 dogs enrolled in the ATR-101 clinical trial

Dog	Age (yrs)	Sex	Breed	Weight	Etiology	Protocol
1	9	M/N	Boxer	36.2 kg	pituitary	2 weeks
2	12	F/S	Lab/Chow	46.0 kg	adrenal	2 weeks
3	9	M/N	Mix	24.5 kg	pituitary	2 weeks
4	8	F/S	Labrador	44.0 kg	pituitary	2 weeks
5	12	M/N	Shi Tzu	8.9 kg	pituitary	4 weeks
6	13	F/S	American Eskimo	19.1 kg	adrenal	4 weeks
7	11	F/S	Labrador	24.0 kg	pituitary	4 weeks
8	15	F/S	Pekingese/Poodle	8.2 kg	adrenal	4 weeks
9	8	M/I	Pitbull	31.0 kg	pituitary	4 weeks^a^
10	9	M/I	Siberian Husky	34.0 kg	pituitary	4 weeks

The 10 dogs were middle-aged to older dogs with an equal distribution of males and females, similar to what has been reported previously (Melián G et al., 2010). Etiology was determined based on results of abdominal ultrasound examinations and measurements of endogenous ACTH concentrations. Protocol refers to the length of treatment with ATR-101

N, neutered; S, spayed; I, intact.

^a^Dog 9 was withdrawn from the study at day 24 due to complications from an unrelated prostatic carcinoma. This dog still was utilized in the study as clinical signs (polyuria and polydipsia, polyphagia, pot-bellied appearance, alopecia, muscle wasting), laboratory test results (stress leukogram, increased alkaline phosphatase activity, hypercholesterolemia, urine specific gravity of 1.006), and pre- (226 nmol/L; reference interval, 15–115) and post-ACTH-stimulated (1272 nmol/L; reference interval, 220–550 nmol/L) cortisol concentrations provided clear and convincing evidence for CS at the time of enrollment

### ATR-101 pharmacokinetics

**Fig. 1:**
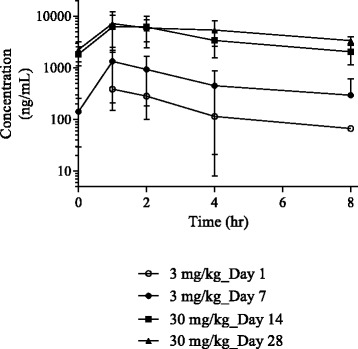
Serum drug concentration vs. time curves following oral administration of ATR-101 to dogs with Cushing’s syndrome. Solid symbols represent steady state concentrations. Values are shown as mean ± SD for *n* = 4 (day 1), 10 (days 7 and 14), and 5 (day 28)

Pharmacokinetics of ATR-101 at both 3 mg/kg and 30 mg/kg are summarized in Table 2. Overall, drug exposures were greater with increased dose (Fig. 1). There appears to be saturation of extravascular clearance (CL/F) and/or a change in bioavailability between day 1 and day 7. Drug accumulation between day 1 and day 7 was observed while accumulation at day 30 was modest. Changes in apparent (extravascular) T_1/2_ were observed at 30 mg/kg after 7–14 days of once daily dosing. Increases in steady state exposure (AUC_0–24_) after repeat daily dosing at 3 and 30 mg/kg appeared dose proportional, within the observed variability, with a 8.2-fold increase in AUC_0–24_ for a 10-fold increase in dose. The corresponding increases in C_max_ were less than dose proportional with a 4.5-fold increase in C_max_ for a 10-fold increase in dosage from 3 mg/kg to 30 mg/kg. The increases AUC_0–24_ and C_max_ were significant (*P* < 0.001). Half-life and Vz/F at steady-state appeared to increase as the dosage was increased from 3 to 30 mg/kg; however, these changes were not significant (*P* > 0.05). Oral clearance did not change as the dosage was increased from 3 to 30 mg/kg (*P* > 0.05).

**Table 2:** Pharmacokinetics parameters in dogs following once daily oral administration of ATR-101

Dose (mg/kg)	Day	Animal number	T_1/2_ (hr)	T_max_^a^ (hr)	C_max_ (ng/mL)	AUC_0–8_ (hr*ng/mL)	AUC_0–24_ (hr*ng/mL)	AUC_0-∞_ (hr*ng/mL)	CL/F (mL/hr./kg)	V_z_/F (mL/kg)
3	1	*n*	3	4	4	4	3	3	3	3
		Mean	2.76	1.0	445	1240	1050	1040	3440	15,600
		SD	1.04	1.0, 4.0	159	697	462	455	1960	14,500
3	7	*n*	4	10	10	10	7	4	7	4
		Mean	2.78	1.0	1480	4560	5790	5050	859	3600
		SD	0.196	1.0, 4.0	1100	3180	4060	3170	665	2810
30	14	*n*	5	10	10	10	5	5	5	5
		Mean	6.56	2.0	6790^***^	29,900	47900^***^	53,700	706	6360
		SD	4.75	1.0, 4.0	4350	15,900	20,000	24,500	252	3640
30	28	*n*	2	5	5	5	4	2	4	2
		Mean	9.36	1.0	9000	39,100	62,500	66,800	497	8370
		SD	NR	1.0, 4.0	3540	11,000	12,200	NR	119	NR

All values are expressed as mean ± SD. with the exception of T_max_. Three dogs had 24 h post-dose data reported and utilized in the analysis. AUC_0-∞_ is utilized in CL/F and V_z_/F calculations on day 1 while AUC_0–24_ (e.g., AUC_0-tau_, tau = dosing period) is utilized in CL/F and V_z_/F calculations at steady state (e.g., day, 7, day 14, day 28). T_max_ as well as pharmacokinetic parameters on days 1 and 28 were not evaluated statistically due to limited sample sizes. T_1/2_, elimination half-life; T_max_, time to maximum serum concentration; C_max_, maximum serum concentration; AUC, area under the serum concentration-time curve; CL/F, apparent total serum clearance after oral administration; V_z_, Apparent volume of distribution during terminal phase after oral/extravascular administration

^a^Median and range (Min, Max) are presented

***P < 0.001 versus day 7 by a two-tailed Student’s *t*-test

### Effects of ATR-101 on basal and ACTH-stimulated plasma cortisol and aldosterone concentrations

**Fig. 2:**
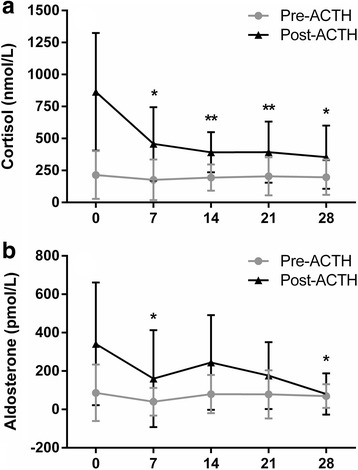
Effects of ATR-101 treatment on (**a**) pre- and post-ACTH-stimulated cortisol and (**b**) aldosterone concentrations in dogs with Cushing’s syndrome. ATR-101 was administered as an oral dose at 3 mg/kg (day 1–7) and 30 mg/kg (day 8–28). Data are given as mean ± SD for *n* = 10 (days 0, 7 and 14), 6 (day 21) and 5 (day 28). ATR-101 administration decreased ACTH-stimulated cortisol concentrations by day 7, an effect that was maintained throughout study duration. Increasing ATR-101 dose on day 8 did not result in further reductions in cortisol concentrations. Aldosterone concentrations fluctuated throughout the study and were decreased as compared to baseline on days 7 and 28. No effects were observed on pre-ACTH stimulated cortisol or aldosterone concentrations. *P < 0.05 versus baseline, **P < 0.01 versus baseline by repeated measures analysis of variance followed by a Dunnett’s post hoc test

The post-ACTH-stimulated cortisol concentrations (reference interval for normal dogs; 220 – 550 nmol/L) at days 7, 14, 21, and 28 were decreased compared to the baseline (day 0) post-ACTH-stimulated cortisol concentration (*P* < 0.05, Fig. 2a). Overall, reductions in post-ACTH-stimulated cortisol concentrations were observed in 9 of 10 dogs, including 7 of 7 dogs with pituitary-dependent CS and 2 of 3 dogs with adrenal-dependent CS. In dogs with adrenal-dependent CS, the post-ACTH-stimulated cortisol concentrations at day 14 were increased by 43.2% in one dog (dog 2) and decreased by 36.1 and 84.2% in the other two dogs as compared to baseline (day 0) concentrations. In dogs with pituitary-dependent CS, the mean ± SD percent reduction in post-ACTH-stimulated cortisol concentrations at day 14 was 49.8 ± 12.8% as compared to baseline concentrations. Post-ACTH-stimulated cortisol concentrations were not different between dogs with adrenal- or pituitary-dependent disease (*P* > 0.05). The mean baseline pre-ACTH-stimulated cortisol concentration (reference interval for normal dogs; 15 – 110 nmol/L) was not different than mean pre-ACTH-stimulated cortisol concentrations after treatment with ATR-101 at either dose (*P* > 0.05); however, 4 of 10 individual dogs did experience reductions. Post-ACTH-stimulated aldosterone concentrations (reference interval; 197 – 2103 pmol/L) were decreased following treatment with ATR-101 at days 7 and 28 (*P* < 0.05, Fig. 2b); however, no effect was observed on pre-ACTH-stimulated aldosterone concentrations (*P* > 0.05) (reference interval; 14 – 957 pmol/L).

### Effects of ATR-101 administration on routine hematologic and serum biochemical parameters

Serum alanine aminotransferase (ALT) activity increased over time, reaching significance on day 28 (*P* < 0.05, Table 3). A similar, albeit non-significant (*P* > 0.05), trend in alkaline phosphatase (ALP) activity was observed with 8 of 10 dogs experiencing a 2 to 4 times increase in ALP activity by study completion. Mild reductions in hematocrit and serum albumin concentration also were observed (*P* < 0.05), although they remained within reference intervals throughout the study. Hematocrit rebounded on day 28, and serum albumin stabilized on day 28. Other hematologic and serum biochemical parameters, including serum sodium and potassium concentrations, did not change, and no dog in the study developed hyponatremia or hyperkalemia despite some changes in post-ACTH-stimulated aldosterone concentrations. The mean ± SD urine specific gravity of 1.016 ± 0.014 at baseline evaluation was not different from urine specific gravities of 1.018 ± 0.015 and 1.023 ± 0.019 at study completion on days 14 and 28 (*P* > 0.05).

**Table 3:** Results of selected serum biochemical parameters in 10 dogs with Cushing’s syndrome treated with orally administered ATR-101

	Reference	Day 0	Day 7	Day 14	Day 21	Day 28
Hct	41–55 (%)	52.4 ± 5.7	50.3 ± 6.6^*^	49.0 ± 6.2^***^	44.2 ± 8.3^**^	47.6 ± 5.1
Bili	0.1 – 0.4 (mg/dL)	0.19 ± 0.07	0.23 ± 0.05	0.17 ± 0.07	0.20 ± 0.00	0.14 ± 0.05
Alb	2.8 – 4.0 (g/dL)	3.11 ± 0.47	3.07 ± 0.42	2.95 ± 0.43^***^	2.82 ± 0.52^**^	2.82 ± 0.48^**^
ALT	14 – 102 (U/L)	136.7 ± 155.4	145.5 ± 142.4	236.7 ± 316.6	362.5 ± 331.1	445.6 ± 340.0^*^
ALP	13 – 107 (U/L)	1025 ± 1633	1172 ± 1886	2121 ± 3928	4113 ± 6487	4151 ± 6555
Chol	124 – 343 (mg/dL)	373.6 ± 133.8	375.1 ± 121.8	389.1 ± 147.7	340.3 ± 151.3	322.0 ± 163.4
SUN	5 – 34 (mg/dL)	17.6 ± 6.2	16.7 ± 8.9	17.8 ± 6.6	20.8 ± 7.1	21.4 ± 9.0
Na	143 – 149 (mmol/L)	148.5 ± 1.6	146.8 ± 1.9	146.3 ± 2.5	146.3 ± 1.4	146.4 ± 1.9
K	3.4 – 5.2 (mmol/L)	4.9 ± 0.6	4.6 ± 0.5	4.6 ± 0.4	4.5 ± 0.6	4.7 ± 0.8
Cr	0.7 – 2.0 (mg/dL)	0.9 ± 0.3	0.9 ± 0.3	0.9 ± 0.3	1.0 ± 0.3	1.1 ± 0.4
Glu	80 – 120 (mg/dL)	99.3 ± 9.8	104.0 ± 13.4	98.4 ± 8.2	94.7 ± 8.9	94.4 ± 11.6

All value are expressed as mean ± SD for 10 dogs (days 0 – 14), 6 dogs (day 21), and 5 dogs (day 28)

Hct, hematocrit; Bili, bilirubin; Alb, albumin; ALT, alanine aminotransferase; ALP, alkaline phosphatase, Chol, cholesterol; SUN, serum urea nitrogen; Na, sodium; K, potassium; Cr, creatinine, Glu, glucose

*P < 0.05 versus day 0; **P < 0.01 versus day 0; ***P < 0.001 versus day 0 by repeated measures analysis of variance followed by Dunnett’s post hoc test

### Evaluation of clinical effects

Owners of the 10 enrolled dogs maintained daily medication logs and documented the presence or absence of potential adverse effects, including vomiting, diarrhea, anorexia, and worsening weakness (if present initially). Owners of all dogs reported that the medication was well-tolerated. Overall, out of 220 study days for the 10 dogs, 4 episodes of vomiting (dogs 6, 7, and 8) and one episode of diarrhea (dog 8) were reported. Three of 4 vomiting episodes were greater than 5 h after drug administration. Clinical signs related to CS were reported as improved in 7 dogs, unchanged in 2 dogs, and worsened in 1 dog. This included improved energy and activity level (dogs 1, 3, 6, 9 and 10), lessening polyphagia (dogs 6, 7, 9 and 10), lessening polyuria and polydipsia (dogs 7, 9, and 10), and improved dermatologic lesions (dogs 4 and 10). Dog 2 experienced worsening polyuria, polydipsia, and polyphagia and was the only dog in this study in which reductions in post-ACTH-stimulated cortisol concentrations were not observed. Dog 9, which showed clinical improvement during the first two weeks, experienced lethargy, weakness, and anorexia in the 4th week, and ATR-101 treatment was discontinued on day 24. Clinical signs persisted and the dog was euthanized two weeks later. Based on the necropsy evaluation, which revealed numerous pulmonary metastases from a known prostatic carcinoma, the clinical signs in this dog were not thought to be related to ATR-101.

### Histologic characterization of adrenal tissue in 3 dogs with cortisol secreting adrenocortical lesions

**Fig. 3:**
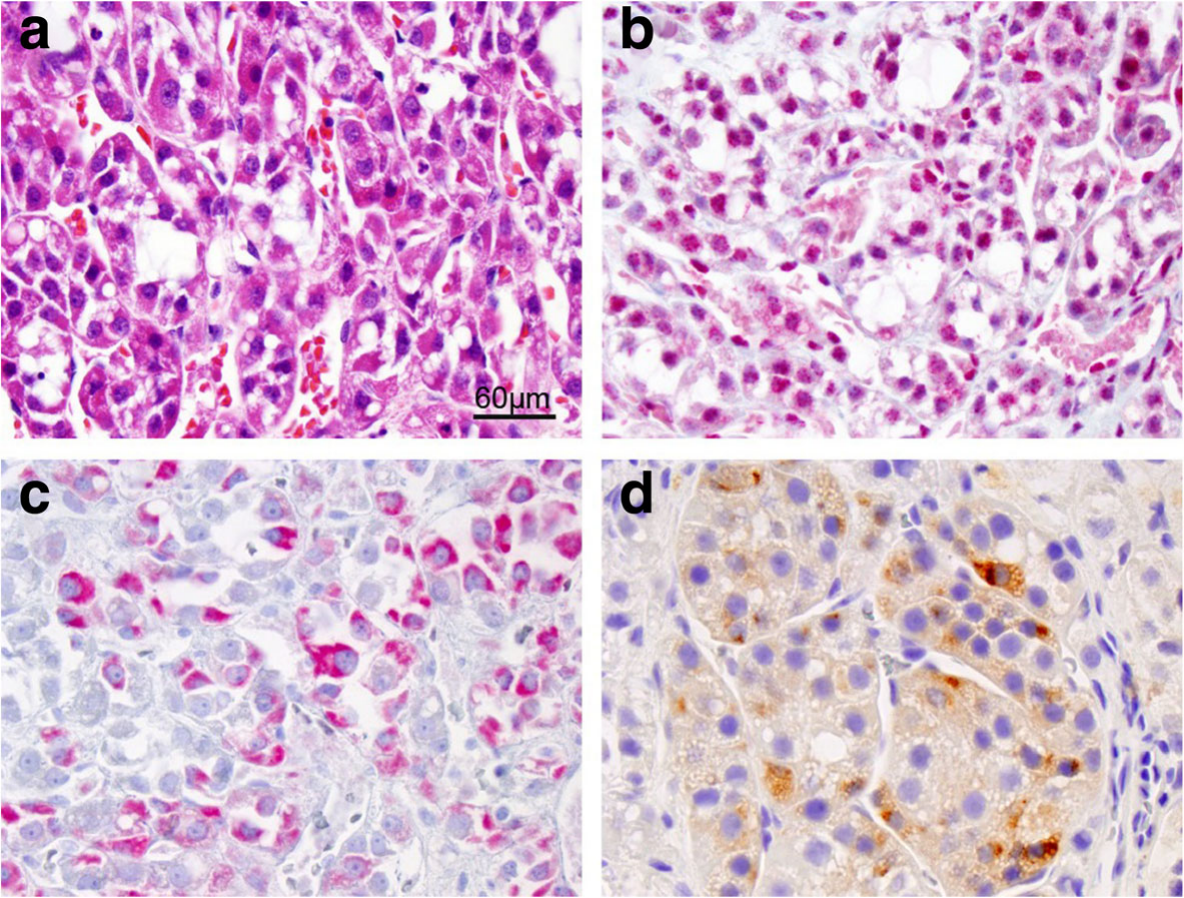
Histologic approach used to evaluate adrenal tissue in dogs with adrenal-dependent Cushing’s syndrome treated with ATR-101. (**a**) H&E stained adrenal tissue demonstrating proliferative neoplastic adrenocortical cells arranged in packets separated by fine fibrovascular stroma in a dog with adrenocortical carcinoma (40×, bar = 60 μm); (**b**) strong nuclear immunoreactivity for caspase-3 indicating increased cellular apoptotic activity (Vector red chromogen, 40×); positive cytoplasmic immunolabeling for Melan-A (Vector red chromogen, 40×) (**c**) and inhibin (diaminobenzidine chromogen, brown, 40×) (**d**) confirming adrenocortical origin

Adrenocortical lesions (Fig. 3) were confirmed in 3 dogs with adrenal dependent disease (dogs 2, 6, 8), including an adrenocortical adenoma (dog 6) and an adrenocortical carcinoma (dog 8). Moderate, multifocal necrosis, fibrosis, granulomatous inflammation, and cholesterol clefts were observed within and surrounding the neoplastic cells in dog 8, while multifocal hemorrhage and mineralization were observed within the adrenal mass in dog 6. In dog 2, histologic sections of tissue were predominantly characterized by marked necrosis, hemorrhage, fibrin accumulation, and granulating fibrosis with mild multifocal dystrophic mineralization. The extent and severity of these lesions in dog 2 precluded accurate classification as adenoma or adenocarcinoma. For dogs 6 and 8, positive immunolabeling for Melan-A (dogs 6, and 8) and inhibin (dog 8) were present in the epithelial cells, confirming adrenocortical origin. Strong nuclear immunoreactivity to caspase-3 was present in adrenocortical cells from dog 8 (80% of cells) while weaker immunolabeling was present in dog 6 (1 – 2% of adrenocortical cells).

## Discussion

Our results document that orally administered ATR-101 reduces post-ACTH-stimulated cortisol concentrations in dogs with naturally occurring CS. This effect was observed by day 7 of ATR-101 therapy and sustained through study conclusion on day 28. Mild reductions of aldosterone concentrations were observed, but this effect was not thought to be clinically relevant as plasma aldosterone and serum electrolyte concentrations remained normal for the study duration. The minimal adverse effects also compare favorably with the adverse effect profiles of some currently used drugs such as mitotane. Positive hormonal (cortisol) effects were observed in all dogs with pituitary-dependent disease and 2 of 3 dogs with adrenal-dependent disease. These findings are encouraging given current limitations of medical therapy for CS in humans and the poor survival times and the difficulties in medically controlling hypercortisolemia in both humans and dogs with malignant adrenocortical lesions [16, 17, 19, 20, 39, 40]. The histologic changes in adrenal glands from dogs with adrenal-dependent disease further support previous findings in healthy dogs that ATR-101 distributes to the adrenal glands, inhibits adrenal ACAT1 activity, and induces apoptosis [25, 27]. Although this was not a blinded and controlled study, clinical improvements were reported by the majority of dog owners. In the aggregate, these results are supportive of further investigations and the ongoing development of ATR-101.

A limitation hampering human CS drug development is the paucity of disease and the failure to identify a naturally occurring model of disease. Although reported incidence varies, CS is rare in humans with an approximate annual incidence of 2 cases per million per year [41]. If CS is rare, adrenocortical carcinoma is exceedingly rare with approximately 600 new cases diagnosed in the Unites States each year [17]. In contrast, canine CS is a common endocrinologic disorder occurring at an estimated incidence of 1–2 cases per 1000 dogs per year, representing a thousand-fold greater incidence as compared to humans [42]. Despite disparity in disease frequency, the etiology, clinical signs, and biochemical and histologic characteristics are remarkably similar between species [2, 3, 43]. Furthermore, dogs age approximately 5–7 times faster than humans, and progression of CS is also accelerated. These features make the dog appealing for both molecular studies and clinical trials. Although this potential has been recognized by others [1, 4, 43], the clinical trial reported herein is the first study using naturally occurring CS in the dog to aid in clinical proof of concept for human CS. This should establish a valuable precedent in using the dogs with naturally occurring CS for the development of human therapeutics, hopefully resulting in benefits for both species.

Although one goal in both humans and dogs is to reduce circulating cortisol concentrations and alleviate clinical signs associated with this excess, methods for monitoring medical therapy can differ [2, 16, 20, 31]. Reductions in pre-ACTH-stimulated (basal) cortisol levels were observed in 4 dogs following ATR-101 therapy, but overall changes were not significant. The exact reasons for this are unclear, but are likely related to the episodic patterns of cortisol secretion observed in dogs that are different from the circadian or diurnal patterns of cortisol secretion observed in other species [44, 45]. Cortisol levels in normal dogs can decrease below and increase above established resting intervals throughout the day. High variability in basal or non-ACTH-stimulated cortisol levels is also observed in dogs with CS [46]. Because of this, measurements of unstimulated cortisol concentrations are not of great utility in the diagnosis or monitoring of CS in dogs [47]. While repeated 24 h urinary free cortisol determinations are routinely used for monitoring CS therapy in humans, this is impractical for most pet owners [6]. The urine cortisol to creatinine ratio, the most analogous clinical test in veterinary medicine, does not provide a consistently correct assessment of hormonal status for therapeutic monitoring of dogs with CS [20, 48, 49]. As such, the ACTH stimulation test is the current standard for monitoring medical therapy in dogs, and this was the basis for our decision to use this test for monitoring response to ATR-101 [20]. Reductions in 24 h urinary cortisol in humans and reductions in post-ACTH-stimulated cortisol concentrations in dogs do correlate with clinical improvement, and it would be logical to assume that reductions in one monitoring method would be accompanied by reductions in the other. However, one limitation of the current study is that it is unknown how well these monitoring methods correlate with each other. Given that pre-ACTH-stimulated cortisol level do not necessarily correlate to the degree of hypercortisolemia throughout the day, additional monitoring methods might prove beneficial. Although the urine cortisol to creatinine ratio is not as effective as ACTH stimulation testing for monitoring efficacy of medical therapy in dogs, values are known to decrease from baseline values following treatment with trilostane or mitotane [48, 49]. This monitoring method should be considered in future studies to enhance the translational aspect as it is most analogous to 24 h urinary free cortisol determinations utilized in humans. Other potential assessments of the degree of non-ACTH-stimulated hypercortisolism such as salivary, hair, or fecal cortisol measurements have undergone minimal investigation in dogs with CS, and their role in therapeutic monitoring is unknown [50–52]. In addition to providing another measure of efficacy, these methods might serve as a more direct correlate to the repeated 24 h urinary free cortisol measurements utilized in humans. Future studies in which dogs with naturally occurring CS are used as a human CS model should consider incorporating multiple treatment monitoring methods, including an assessment of resting hypercortisolism.

In the current study, reductions in post-ACTH-stimulated cortisol concentrations were accompanied by subjective owner-reported clinical improvements in many dogs, although complete symptom resolution was not achieved in any of the dogs. The reasons for incomplete symptom resolution are likely two-fold. First, many clinical signs, especially physical changes and dermatologic abnormalities, can take several months to resolve [20]. Second, the observed reductions in post-ACTH-stimulated cortisol concentrations were heterogeneous and not of the magnitude typically associated with resolution of clinical signs [20, 31]. The lack of further decreases in cortisol concentrations despite an increased ATR-101 dose commencing on day 8 is in contrast to a previous study of ATR-101 in healthy dogs in which dose-dependent decreases in ACTH-stimulated cortisol concentrations were observed [27]. The reasons for the heterogeneity of post-ACTH cortisol responses and the discrepancy between studies are unclear; however, it is plausible that adrenocortical ACAT1 expression could differ between normal dogs and dogs with CS, or even among individual dogs with CS [28]. Similarly, the utilization of only 2 set dosages administered at only 1 frequency in dogs with varying types and severities of CS may account for some variation. The timing of ACTH stimulation testing may also be important as the optimal time for testing in trilostane treated dogs is 4 to 6 h post-drug administration [20]. Regardless, medical therapy of CS in dogs is often individualized as variable dosages and dosing frequencies are utilized in clinical settings [14]. Extended treatment duration, higher ATR-101 dose, or more frequent dosing potentially could result in further hormonal and clinical improvement. Additional studies are needed to evaluate these hypotheses.

Clinical signs of liver disease were not observed in the current study, and biochemical parameters associated with liver function such as bilirubin, glucose, and urea nitrogen remained normal. However, the increases in liver enzyme activity are noteworthy. Dogs with CS usually have increased liver enzyme activity at diagnosis [3], but the continual increases over time despite reductions in post-ACTH-stimulated cortisol concentrations were unexpected. ACAT1 is known to be expressed in numerous tissues including Kupffer cells within the liver; however, expression of ACAT1 is markedly higher in the adrenal cortex which is likely one reason for the adrenal selective effects of ATR-101 [27, 53]. In a previous in-vitro study, ATR-101 did not induce toxic effects on hepatocytes unless the cells were pretreated with agents to block glycolysis or inhibit cytochrome P450-mediated metabolism [54]. In an unpublished toxicity study conducted by two authors of this report (MB and SH), ATR-101 administration to healthy dogs resulted in mild increases in ALP and ALT activities. Histologically, non-glycogen, non-lipid containing vacuolar hepatic change was observed which was not accompanied by necro-inflammatory activity. Both abnormal enzyme activity and histologic changes resolved upon discontinuation of ATR-101. Given that ATR-101 is metabolized by the liver, the increased enzyme activity likely represents an adaptive response to hepatic drug metabolism [55], but the cause for these changes was not determined. The reported reductions in hematocrit in dogs from our study are unlikely to be of clinical significance as they remained within normal reference intervals for the study duration and rebounded by study completion. The reductions in albumin were minor and not deemed to be related to ATR-101 administration or clinically relevant as they also remained within normal reference intervals and appeared to plateau by study completion.

## Conclusions

ATR-101 is well-tolerated and reduces post-ACTH-stimulated cortisol concentrations in dogs with naturally occurring CS. Given the striking similarities between CS in dogs and humans, naturally occurring canine CS appears to be a suitable model for human studies. The results reported herein further support the ongoing development of ATR-101 for treatment of disorders associated with excess adrenal steroid production in humans, such as CS, congenital adrenal hyperplasia, and ACC. Additional investigations are required to optimize dosing strategies and further evaluate the effects of prolonged drug administration on efficacy and safety.

## Abbreviations

ACC: Adrenocortical carcinoma
ACTH: Adrenocorticotropic hormone
ALP: Alkaline phosphatase
ALT: Alanine aminotransferase
AUC: Area under the serum drug concentration-time curve
CS: Cushing’s syndrome
LDDST: Low-dose dexamethasone suppression test
